# Paxillin tunes the relationship between cell–matrix and cell–cell adhesions to regulate stiffness-dependent dentinogenesis

**DOI:** 10.1093/rb/rbac100

**Published:** 2022-12-10

**Authors:** Mingru Bai, Zhaowei Zhang, Huiyu Chen, Xiaoyu Liu, Jing Xie

**Affiliations:** State Key Laboratory of Oral Diseases & National Clinical Research Center for Oral Diseases, West China Hospital of Stomatology, Sichuan University, Chengdu, China; Department of Endodontics, West China Hospital of Stomatology, Sichuan University, Chengdu 610041, China; State Key Laboratory of Oral Diseases & National Clinical Research Center for Oral Diseases, West China Hospital of Stomatology, Sichuan University, Chengdu, China; State Key Laboratory of Oral Diseases & National Clinical Research Center for Oral Diseases, West China Hospital of Stomatology, Sichuan University, Chengdu, China; State Key Laboratory of Oral Diseases & National Clinical Research Center for Oral Diseases, West China Hospital of Stomatology, Sichuan University, Chengdu, China; State Key Laboratory of Oral Diseases & National Clinical Research Center for Oral Diseases, West China Hospital of Stomatology, Sichuan University, Chengdu, China

**Keywords:** mechanotransduction, cell–cell contacts, cell–substrate adhesions, odontogenic differentiation

## Abstract

Mechanical stiffness is recognized as a key physical factor and directs cell function via a mechanotransduction process, from extracellular physical cues to intracellular signaling cascades that affect transcriptional activity. Cells continually receive mechanical signals from both the surrounding matrix and adjacent cells. However, how mechanical stiffness cue at cell–substrate interfaces coordinates cell–cell junctions in guiding mesenchymal stem cell behaviors is poorly understood. Here, polydimethylsiloxane substrates with different stiffnesses were used to study mechanosensation/transduction mechanisms in controlling odontogenic differentiation of dental papilla cells (DPCs). DPC phenotypes (morphology and differentiation) changed in response to the applied force derived from stiff substrates. Significantly, higher expression of paxillin on stiffer substrates promoted DPC dentinogenesis. Upon treatment with siRNA to knockdown paxillin, N-cadherin increased mainly in the cytomembrane at the area of cell–cell contacts, whereas β-catenin decreased in the nuclei. The result of a double luciferase reporter assay showed that stiffness promoted β-catenin binding to TCF, which could coactivate the target genes associated with odontogenic differentiation, as evidenced by bioinformatics analysis. Finally, we determined that the addition of a β-catenin inhibitor suppressed DPC mineralization in all the stiffness groups. Thus, our results indicated that a mechanotransduction process from cell–substrate interactions to cell–cell adhesions was required for DPC odontogenic differentiation under the stimulation of substrate stiffness. This finding suggests that stem cell fate specification under the stimulus of stiffness at the substrates is based on crosstalk between substrate interactions and adherens junctions, which provides an essential mechanism for cell-based tissue engineering.

## Graphical abstract

**Figure:**
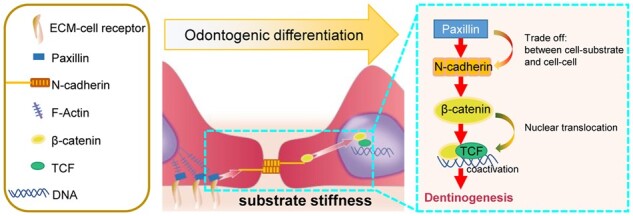

## Introduction

The extracellular matrix (ECM) is mainly composed of polysaccharides and fibrillar proteins for anchoring cells and controlling cell behaviors by providing biochemical cues as well as physical stimuli [1, 2]. Among them, mechanical cues play a key role in directing cell function via a mechanotransduction process. Through mechanosensing and mechanotransducing pathways, cells integrate mechanical signals from the surrounding ECM and transduce them into intracellular biological molecular signals to direct phenotypes [3]. Typically, this step regulates focal adhesion (FA) formation and cytoskeleton assembly, which further triggers signaling cascades that impact transcriptional activity.

ECM stiffness critically dictates cell behaviors. Revealing cell responses to different stiffnesses can direct biomaterial design to orchestrate cell function in tissue regeneration [4]. Over the past few decades, stiffness-mediated mechanical pathways have been widely reported. However, most of these studies have revealed the mechanosignaling pathway from the ECM stiffness to the nucleus in a single cell. Mammalian cells are surrounded by not only the ECM but also neighboring cells [5]. Cells continuously capture and integrate mechanical signals from both the surrounding ECM and adjacent cells, indicating that mechanical signal transmission is complex. To the best of our knowledge, few studies have reported the role of mechanical stiffness signal transduction—initiating from cell–material interfaces to cell–cell adhesions—in guiding cell differentiation. Therefore, studies on stiffness-dependent mechanotransduction from cell–substrate contacts to intercellular adhesions are required to sufficiently track mechanical signals in complicated molecular networks.

Cell–ECM interactions (FAs) or cell–cell interactions (cadherin junctions) contribute to adhesion or contractile force in the same cytoskeletal network, causing intracellular signaling cascade reactions [6, 7]. Thus, the relationship between cell–ECM and cell–cell adhesions was considered a trade-off: strong cell–ECM contacts weakened cell–cell junctions [8]. Cells receive mechanical cues at cell–material interfaces, which enables conformational changes in FAs. FAs, force-sensitive protein complex structures, are designated as mediators between cells and substrates [9, 10]. Paxillin is a multidomain focal adhesive protein that localizes primarily to FAs [11] and serves as a point of convergence for signal transduction [12]. Adherens junctions are formed by cadherin-based adhesive contacts at the intercellular neighboring interface [13]. Cadherins are a multigene family of Ca^2+^-dependent glycoproteins that engage in cell–cell adhesions [14]. Importantly, they have also been reported to propagate intracellular signaling cascades to influence cellular behaviors in different cell types [15–17]. Structured illumination microscopy images and intensity profile analysis demonstrated that paxillin overlapped with cadherin [18]. However, it is unclear whether the relationship between paxillin and N-cadherin is synergistic or competitive. Herein, we hypothesized that the paxillin-regulated N-cadherin/β-catenin complex could be a key point in stiffness-mediated mechanotransduction from cell–substrate adhesions to intercellular junctions during mesenchymal stem cell differentiation.

Dental papilla cells (DPCs) have a multilineage differentiation capacity, contributing for instance, to neurogenesis and osteogenesis [19, 20]. They are appealing candidates in tissue engineering [21], especially in tooth regeneration therapies, because they are derived from the cranial neural crest, and the odontogenic differentiation process follows the cues from their natural function of pulp and dentin formation in tooth germ development *in vivo*. Previous studies have shown that DPCs can be obtained from the human tooth germs of extracted third molars or the developing tooth root apex [22], with the characteristics of highly proliferative, migratory, and regenerative potential. Compared with dental pulp stem cells, stem cells from papilla appear to be more suitable for tissue regeneration with higher proliferative capacity and mineralization potential [23]. Moreover, the easy isolation and large expansion of DPCs facilitates stem cell-based therapy, thereby enabling as a mesenchymal stem cell model for research.

Herein, we aimed to investigate mechanotransduction from cell–material interfaces to cell–cell contacts in the process of DPC odontogenic differentiation in response to mechanical stiffness. Polydimethylsiloxane (PDMS) gels are widely used as the substrates to study mechanotransduction in cells, because of their advantages, including stable chemical properties, transparency, and biological compatibility. The elastic moduli could be effectively changed by varying the density of crosslinking [24]. Thus, we used PDMS gels as cell culture substrates to study cell–substrate interaction. Here, PDMS gels with various elasticities were fabricated to provide a series of stiffnesses for cell culture. First, we evaluated the role of stiffness on DPC morphology and odontogenic differentiation. We then explored the mechanism centered on paxillin at the cell–material interface in response to alterations in stiffness. Finally, we demonstrated that paxillin knockdown modulated the effect of N-cadherin/β-catenin-mediated intercellular force transduction signals, which displayed the mechanotransduction pathway from cell–substrate adhesions to cell–cell junctions via stimulation of PDMS stiffness. Due to the potential of differentiation, dental stem cells take numerous advantages for tooth regeneration. The results revealed that the mechanical properties of microenvironment could regulate DPC behaviors. The study of underlying mechanism provided targets for dental stem cell therapies in cell-based tooth regeneration.

## Materials and methods

### PDMS substrate fabrication

The oligomeric base and Sylgard184 were mixed at decreasing ratios (Sylgard184/oligomeric base = 1:5, 1:15, 1:30 and 1:45). Sylgard184 was the curing agent. The mixture was poured onto a Petri dish and allowed to stand overnight. Then the Petri dishes were coated with PDMS. Later, it was heated at 65°C for 12 h. To enhance cell adhesions, dopamine solution was prepared being socked in the tris(hydroxymethyl) aminomethane and dopamine solution (pH 8), which then was coated on PDMS surface for 24 h, twice.

### Physical properties of PDMS substrates

The surface topography was measured using an atomic force microscope (SPM-9700, SHIMADZU, Japan). PDMS surfaces with an area of 10 × 10 mm were measured in the tapping mode. Then, *R_a_* was measured, and the 3D shape of the PDMS surfaces was displayed. The stress–strain curve was acquired using a universal testing machine, and the elastic modulus was calculated using Origin 2019b.

### Cell culture

DPCs were collected from the tooth germ of C57BL/6 mice on postnatal days 1–3. Experimental mice were sacrificed and sterilized with 75% ethanol. After the mandibles and maxillaries were separated, molar germs were isolated using a mechanical approach. The germ cells were digested with 0.25% trypsin for 20 min and then with 0.2 mg/ml type I collagenase overnight. The collected cells were seeded in Petri dishes in α-MEM (Hyclone, Logan, UT, USA) supplemented with 10% fetal calf serum and 1% penicillin–streptomycin at 37°C in a 5% CO_2_ incubator. For β-catenin inhibition, cells were cultured with 2 µM XAV939 (Apexbio Technology, Houston, USA). The animal experiment was performed with protocols approved by the Institutional Animal Care and Use Committee at State Key Laboratory of Oral Diseases, Sichuan University. The protocol and procedures employed were reviewed and approved by the Research Ethics Committee of West China Hospital of Stomatology.

### Scanning electron microscope

After cultured on different stiffness substrate, cellular sample were fixed with 2.5% glutaraldehyde. Dehydration was used the concentration gradient of ethanol (50%, 70%, 80%, 90% and 100%), each one for 15 min in turn. Samples were scanned after coated with gold.

### Alizarin red staining or calcein staining

DPCs were seeded in a 24-well plate and cultured in a conditioned medium to induce mineralization for 7, 14 and 21 days. The odontogenic medium contained Dulbecco’s modified Eagle’s medium (Hyclone, Logan, UT, USA) supplemented with 10% fetal bovine serum (Hyclone, Logan, UT, USA) and 1% penicillin–streptomycin (Hyclone, Logan, UT, USA), 50 µg/ml ascorbic acid, 10 mM β-glycerophosphate and 0.1 µM dexamethasone. The samples were treated with 2% alizarin red stain or 400 µM calcein stain for 30 min.

### Western blot

Cellular samples were washed twice with PBS. Total proteins were collected by scraping the samples in lysis buffer, followed by concentration detection of the supernatant using bicinchoninic acid protein assay kits. Then, 20–35 µg proteins separation process was performed using 10% SDS-PAGE gels by electrophoresis, and the treated gels were blotted onto polyvinylidene difluoride (PVDF) membranes. The PVDF membranes were incubated with 5% bovine serum albumin (BSA) for 1 h at 25°C. The primary antibodies used were DSPP (Santa Cruz Biotechnology, sc-73632), COL1A1 (ZEN BIO, R26615), alkaline phosphatase (ZEN BIO, 381009), GAPDH (Cell Signaling Technology, 5174S), paxillin (Abcam, ab32084), osterix (Abcam, ab209484), β-catenin (Invitrogen, 13-8400) and N-cadherin (Abcam, ab18203). The primary antibodies were used to incubate the blocked PVDF overnight at 4°C. Horseradish peroxidase-conjugated secondary antibody against goat anti-rabbit or goat anti-mouse antibody was used to incubate the PVDF membrane for 1 h at 25°C. The films were then scanned using a chemiluminescence system.

### Cell immunofluorescence staining

Samples were fixed with 4% pauraformaldehyde for 30 min and blocked with 5% BSA for 1 h at 25°C. After treatment with 0.25% Triton for 10 min, the samples were incubated with primary antibodies. The samples were then treated with Alexa Fluor 647 goat anti-mouse IgG (Abcam, ab150115) or Alexa Fluor 647 donkey anti-rabbit IgG (Abcam, ab150075). The cytoskeleton was stained with phalloidin, and cell nuclei were stained with DAPI. Images were scanned using a confocal microscope (FV3000 and Nikon, A1R MP+, Olympus, Tokyo, Japan).

### Adenovirus infection

Overexpression adenovirus of paxillin was purchased by FUBIO Co., Ltd. (Suzhou, China). The adenovirus (MOI = 100) was used to infect cells for 24 h and then the fresh medium was replaced.

### siRNA transfection

Small interfering RNA (siRNA) was designed with the following sequence: (forward primer: CCUGCAGAUGAAGCCGAAUTT, reverse primer: AUUCGGCUUCAUCUGCAGGTT). After cell confluence reached 70–80% in the cell-culture dishes, they were transfected with siRNA according to the protocol of Lipofectamine™ 3000 Transfection Reagent (Invitrogen, CA, USA). After 6 h, the medium was replaced with a fresh medium. After 48 h, proteins were collected for further analysis.

### RNA sequence

Human dental pulp cells (hDPCs) were seeded onto 1:5 and 1:45 PDMS substrates for 3 days. Total RNA was collected according to the instruction manual of TRIzol™ reagent (Invitrogen, CA, USA) and used for RNA sequence analysis at Beijing Biomarker Technologies Co., Ltd. (Beijing, China) (http://www.biomarker.com.cn). Before sequencing, RNA integrity was evaluated using a Nanodrop 2000 (Thermo Fisher, USA), and RNA concentration was checked using an Agilent 2100 Bioanalyzer (Agilent Technologies, Inc., Santa Clara, CA, USA). In data analysis, the Illumina NovaSeq 6000 platform (San Diego, CA, USA) was used for sequencing. Gene abundance differences were calculated based on the ratio of fragments per kilobase million values. Gene enrichment analyses were performed using KEGG and KOG databases.

### Bioinformatics analysis

Sequences (∼2000 bp) in regions upstream of the transcriptional start sites (the promoter region) of the *Osterix* and *Col1α1* genes were collected from the NCBI web. PROMO was used to predict and analyze specific binding sites. Detailed information is provided in the Supplementary Data.

### Top/FOP flash dual-luciferase reporter assays

Twenty-four-well plates were coated with PDMS and then DPCs were seeded on these PDMS substrates. Cells were treated with 800 ng of TOP flash or FOP flash luciferase reporter plasmid and 20 ng of the pGL4-TK plasmid (Promega, Madison, USA) until their 70–80% confluence was achieved. The transfection was followed by the protocol of Lipofectamine™ 3000 Transfection Reagent (Invitrogen, CA, USA). After 48 h of transfection, cells were extracted, and luciferase activity was evaluated according to the manufacturer’s protocol using the Dual-Luciferase^®^ Reporter Assay System (Promega, Madison, USA). Firefly luciferase was detected using a microplate reader (Thermo Fisher Scientific, Varioskan Flash, USA), and Renilla luciferase was used for normalization.

### Statistical analysis

The statistical significance between groups was determined using Student’s *t*-test or one-way analysis of variance, as appropriate. Results with *P* < 0.05 were considered as statistically significant (*P < 0.05, **P < 0.01, ***P < 0.001). All assays were repeated at least three times.

## Results

### Fabrication of PDMS with tailored stiffness and DPC morphology in response to stiffened surfaces

**Figure 1.**
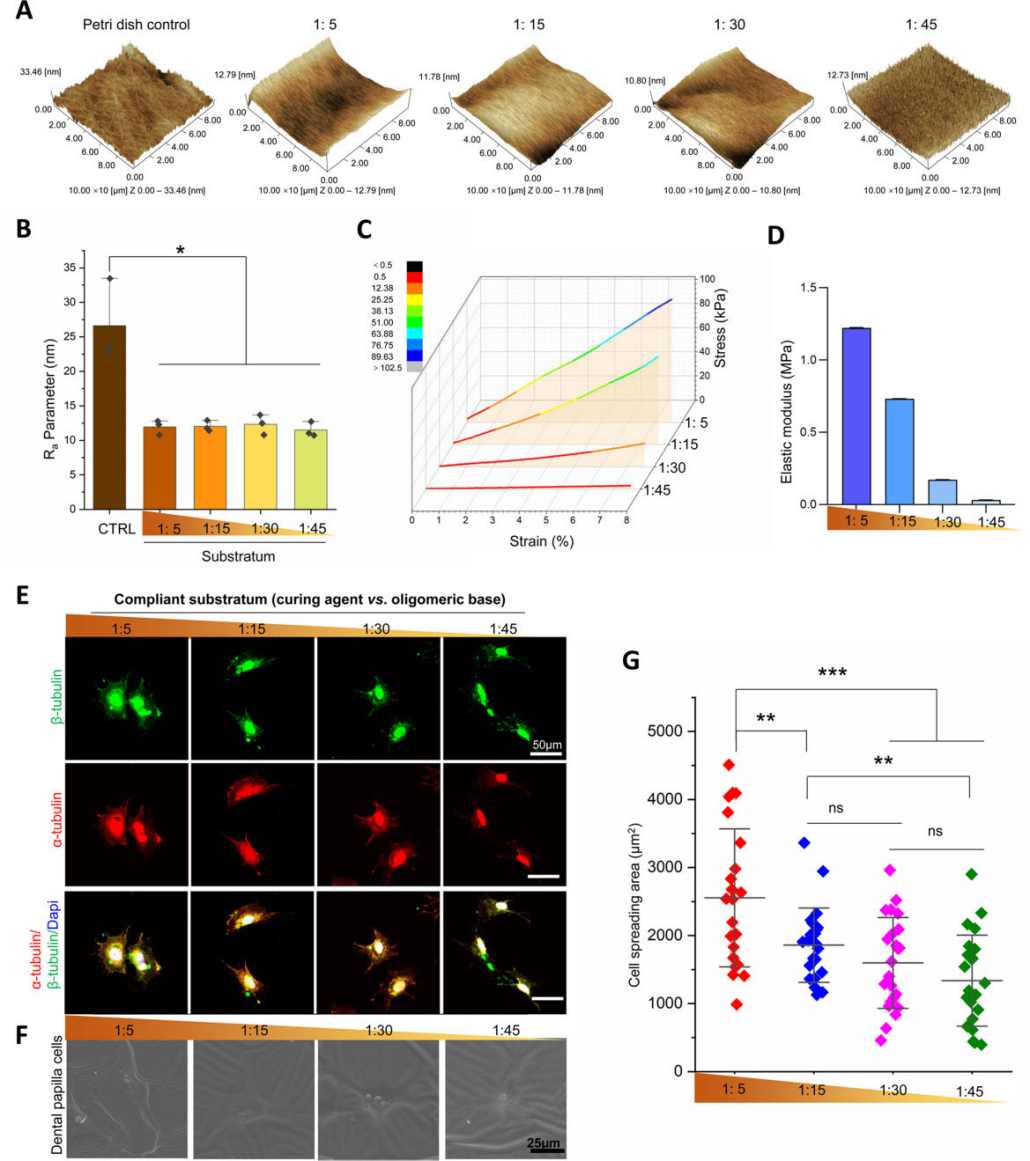
DPC morphological changes on PDMS substrates with different stiffnesses. (**A**) Representative 3D images of Petri dish and PDMS surfaces by AFM. (**B**) *R_a_* values of Petri dish and PDMS substrates according to AFM images (*n* = 3). (**C**) Strain–stress curve of PDMS in different ratios. (**D**) Elastic moduli according to strain–stress curve. (**E**) Representative images of DPC cytoskeleton on different stiffnesses marked by α-tubulin/β-tubulin. (**F**) Representative images of DPC detected by scanning electron microscope. (**G**) Relative quantification of cell spreading area (*n* = 23). Data are means ± SD, *P < 0.05, **P < 0.01, ***P < 0.001.

Four PDMS substrate ratios (curing agent vs. oligomeric base: 1:5, 1:15, 1:30 and 1:45) were fabricated. PDMS with controllable stiffness was coated with dopamine on its surface for cell culture. The surface roughness of the four groups was characterized using atomic force microscopy. The results demonstrated that the *R_a_* parameter was higher on the surface of the Petri dish than on the surfaces of the PDMS substrates (Fig. 1A). The *R_a_* parameters in the four PDMS groups showed no significant differences (Fig. 1B); however, the elastic modulus indicated a decreasing gradient progression from the 1:5 group to the 1:45 group (from 1.22 to 0.03 MPa) (Fig. 1C and D). DPCs were seeded onto the PDMS substrate with different stiffnesses. To investigate tubulin organization, the DPCs were labeled with α-tubulin and β-tubulin. The results showed that most DPCs spread more widely on the stiffer substrate (1:5) and displayed polygonal shapes, whereas they were rounder on the softer substrate (1:45) (Fig. 1E). Scanning electron microscope was used to show the same tendency of cell morphology change (Fig. 1F). The cell-spreading area gradually increased with increasing stiffness (Fig. 1G). Thus, we determined that DPC could respond to increasing stiffness.

### DPC odontogenic differentiation profiles on PDMS substrates with various stiffnesses

**Figure 2.**
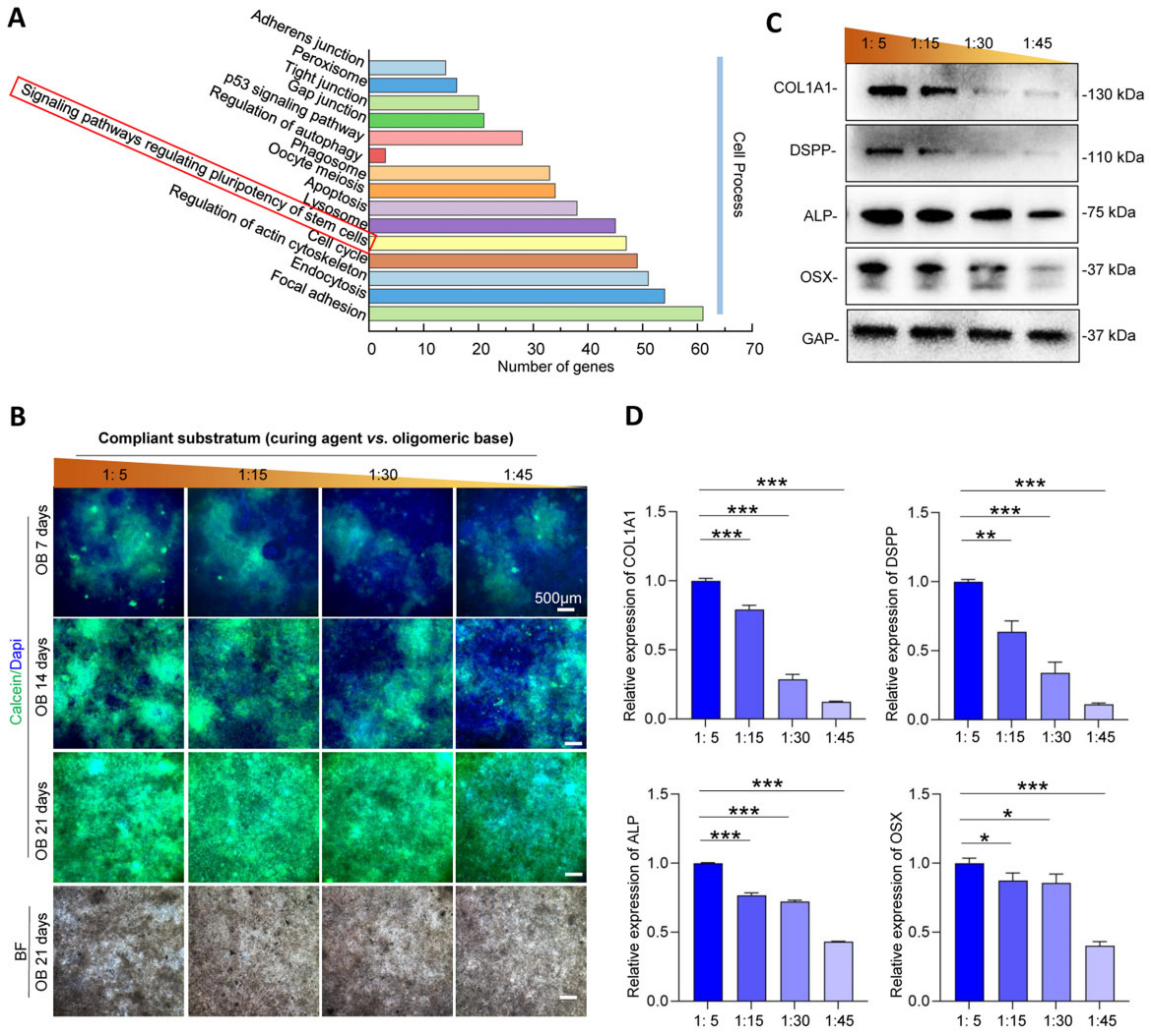
DPC odontogenic differentiation on substrates with different stiffnesses. (**A**) Gene statistics of KEGG classification associated with cell process, comparing the 1:5 (the stiffest) group with 1:45 (the softest) group. (**B**) Calcein staining of mineralized nodule formation after osteogenic induction for 7, 14 and 21 days. (**C**) The representative images of odontogenic markers on PDMS surfaces in different stiffnesses by western blotting. (**D**) Relative quantification of the expression levels of odontogenic markers (*n* = 3). Data are means ± SD, *P < 0.05, **P < 0.01, ***P < 0.001.

After RNA sequence analysis, KEGG classification in the aspect of cell process showed that differential gene expression between the 1:5 group and 1:45 group was associated with ‘signaling pathways regulating pluripotency of stem cells’ (FDR ≤ 0.05 and Log2FC ≥ 1.2) (Fig. 2A). We also assessed multiple biological functions of DPCs on PDMS substrates with different stiffnesses, including odontogenic differentiation, proliferation, apoptosis and migration (Fig. 2B and Supplementary Figs S1–S3). Among them, we considered that the mineralized phenotype of DPCs changed significantly with stiffness. Therefore, we focused on exploring the odontogenic differentiation of DPCs. To reveal the influence of stiffness on the odontogenic differentiation process, the production of extracellular calcium deposits in DPCs was analyzed after odontogenic induction for 7, 14 and 21 days. After the calcified nodules were stained with calcein, the calcein–calcium complexes were observed using a fluorescence microscope. It was observed that higher amounts of calcium deposits were present in DPCs on higher substrates (Fig. 2B). In addition, the protein expression levels of COL1A1, OSX, ALP and DSPP in the DPCs increased with increasing stiffness (Fig. 2C and D). Taken together, these data demonstrated a significant correlation between substrate stiffness and DPC odontogenic differentiation.

### Paxillin mediates stiffness-dependent odontogenic differentiation of DPCs

**Figure 3.**
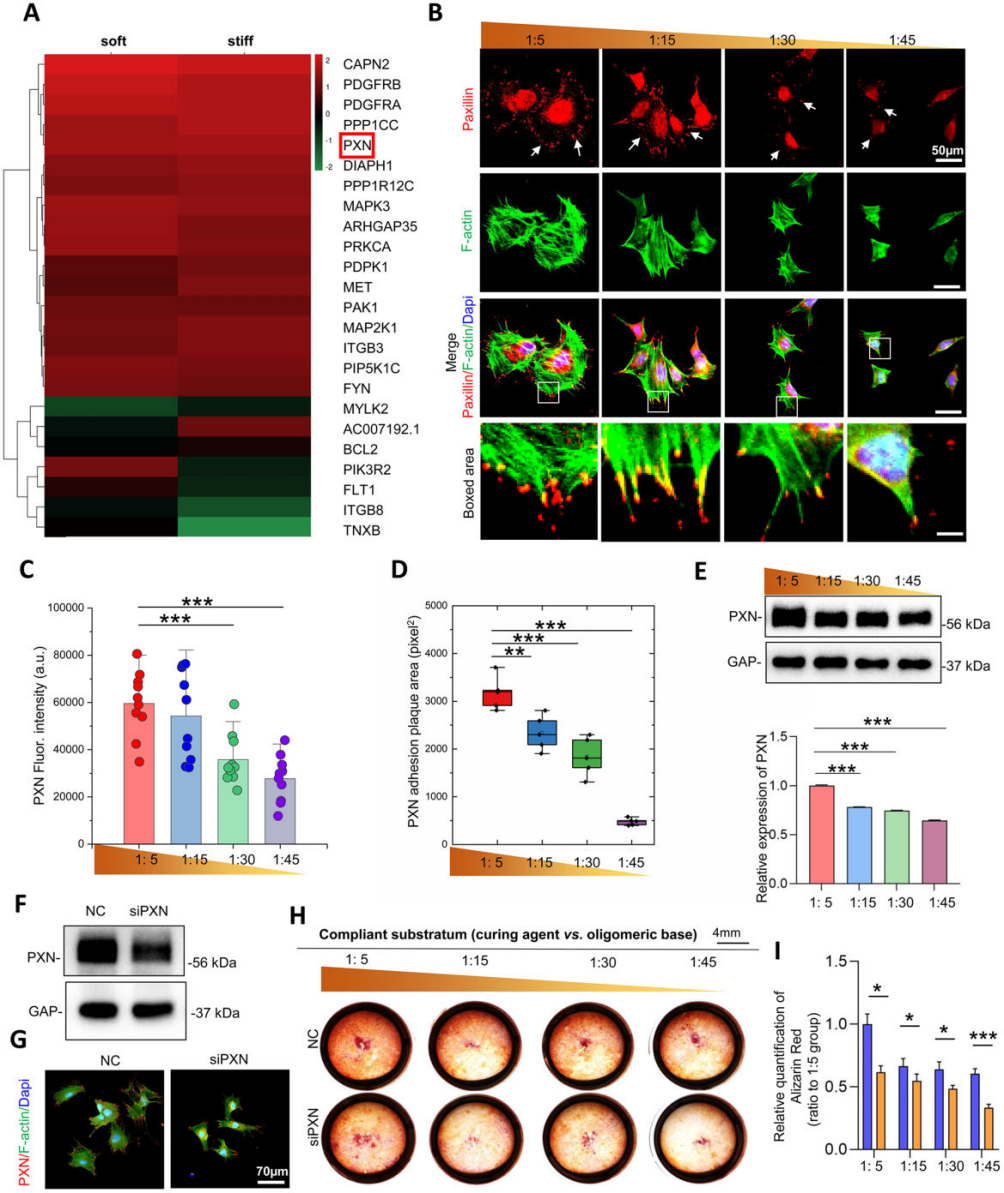
Paxillin expression on different stiffnesses is associated with DPC odontogenic differentiation. (**A**) The results of RNA sequence analysis demonstrated the changes in the top 24 genes involved both in aspect of focal adhesions and signal transduction mechanisms. (**B**) Representative immunofluorescence images of the distribution of paxillin. (**C**) The fluorescence intensity of paxillin. (**D**) The area of the paxillin plaques of DPCs. (**E**) The expression levels of paxillin on substrates with different stiffnesses were detected by western blotting and the relative quantification (*n* = 3). (**F**) After interference with siRNA, total paxillin expression levels in DPCs were detected by western blotting. (**G**) Representative fluorescence images of paxillin distribution with siRNA addition. (**H**) Alizarin red staining of DPCs on PDMS with different rigidity. (**I**) Relative quantification of alizarin red staining. Data are means ± SD, *P < 0.01, **P < 0.05, ***P < 0.001.

RNA sequence analysis revealed that paxillin changed as a FA protein related to signal transduction mechanisms (Fig. 3A). Paxillin is equipped with multiple domains, providing sites for protein localization. Immunofluorescent images of paxillin showed significantly more mature dotted and diffuse expression along the cell margins on stiff substrates, while it displayed immature and smaller spotty patterns in DPCs on soft substrates (Fig. 3B). Significant differences were noted in paxillin fluorescence intensity analysis and in the paxillin adhesion plaque area between the 1:5 and 1:45 groups (Fig. 3C and D). At the protein level, paxillin expression was significantly highest on the stiffest substrate (Fig. 3E).

Cell–ECM adhesions contribute to cellular morphogenesis and behaviors [25]. FAs mediate cell adhesions to the ECM, and they are involved in force transmission and signal transduction. We then determined whether increased paxillin (FA) expression on stiffer substrates was correlated with the enhanced mineralization phenotype in DPCs. To confirm the role of paxillin in stiffness-dependent mineralization, we treated DPCs on substrates of various stiffnesses with small interfering RNA. Knockdown efficiency was determined using western blotting (Fig. 3F). Immunofluorescence staining showed significantly lower expression of paxillin in the cell margins in the siPXN group (Fig. 3G). After 7 days odontogenic induction and paxillin knockdown, the odontogenic differentiation responses of DPCs were detected by Alizarin red staining. The addition of siPXN decreased the mineralization of DPCs in all groups (Fig. 3H and I). Taken together, paxillin mediates the stiffness-dependent mineralization of DPCs.

### Paxillin regulates N-cadherin/β-catenin complex expression and distribution

**Figure 4.**
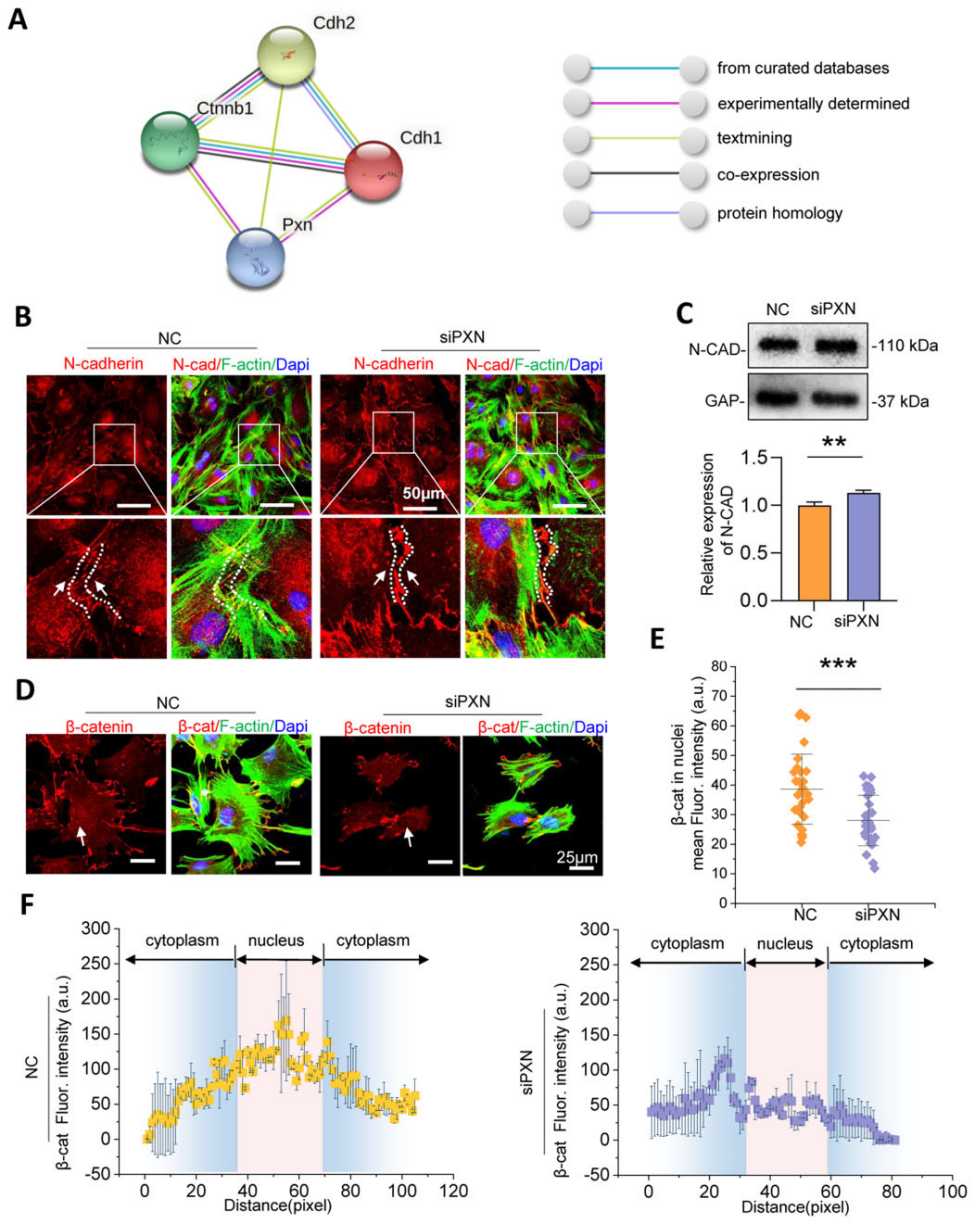
N-cadherin/β-catenin expression and distribution are regulated by paxillin. (**A**) STRING analysis of the relationship between paxillin and N-cadherin (or E-cadherin)/β-catenin. N-cadherin (gene name was cdh2), E-cadherin (gene name was cdh1) and β-catenin (gene name was ctnnb1). (**B**) Representative immunofluorescence images of N-cadherin distribution in DPCs after treatment with siPXN. (**C**) N-cadherin expression levels detected by western blotting after the treatment of siPXN and the relative quantification (*n* = 3). (**D**) Representative immunofluorescence images of β-catenin distribution after treatment with siPXN (*n* = 30). (**E**) The mean fluorescence intensity of β-catenin in nuclei with or without treatment with siPXN. (**F**) The linear profile of β-catenin intensity distribution based on the immunostaining image. Data are means ± SD, **P < 0.01, ***P < 0.001.

To explore this mechanism, we focused on the potential influence of paxillin-based cell–substrate adhesion on intercellular junctions. N-cadherin was a sensor of intercellular mechanics, which mainly expressed in mesenchymal cells. Furthermore, N-cadherin/β-catenin is known as a protein complex [26]. STRING analysis (https://www.string-db.org/) was performed to determine the relationship between paxillin and N-cadherin/β-catenin. The analysis demonstrated that the relationship between PXN and the N-cadherin (or E-cadherin)/β-catenin complex was evaluated by experimental determination and textmining (Fig. 4A). To further investigate the influence of PXN on N-cadherin, we performed immunofluorescence and western blotting, thus confirming the changes in N-cadherin after PXN knockdown. Western blotting showed a slight increase in total N-cadherin (junction molecule) expression in DPCs following treatment with siPXN (Fig. 4C). In addition, we observed that the enhanced expression of N-cadherin was mainly at cell–cell contact areas with PXN knockdown (Fig. 4B), which is the exact binding site between N-cadherin and β-catenin. Considering that β-catenin, a signaling molecule, can enter the nucleus and function as a transcription coactivator, we further explored whether knockdown of paxillin from cell–substrate adhesions led to β-catenin release from the N-cadherin/β-catenin complex and translocation into the nuclei. Immunofluorescence staining was used to assess β-catenin localization. The results demonstrated that β-catenin accumulated less in the nucleus after paxillin knockdown (Fig. 4D–F, Supplementary Fig. S4). Taken together, these observations indicated that intercellular junctions were influenced by mechanical stiffness cues at the cell–substrate contacts. Thus, PXN regulates the distribution of the N-cadherin/β-catenin complex, which provides evidence of mechanotransduction from cell–substrate adhesions to cell–cell junctions.

### Substrate stiffness-induced β-catenin binding to TCF-1 which resulting in DPC odontogenic differentiation

**Figure 5.**
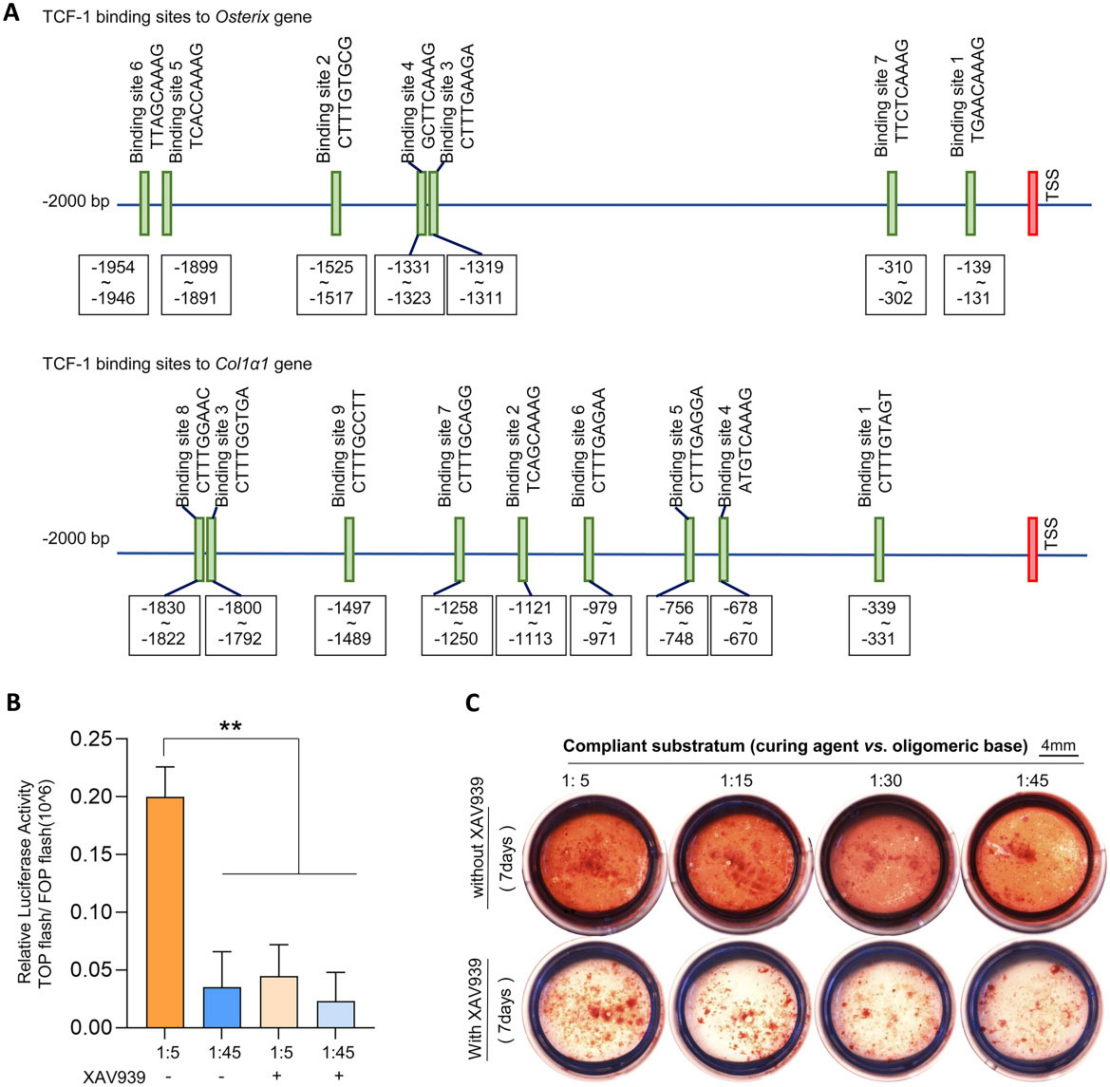
Substrate stiffness-mediated β-catenin transcriptional activity regulating DPC mineralization. (**A**) The bioinformatics analysis of recognized sites of TCF-1 in the promoter of Osterix and Col1a1. (**B**) Relative luciferase activity TOP flash/FOP flash was analyzed by dual-luciferase reporter assay (*n* = 3). (**C**) Alizarin red staining represents calcified formation of DPCs on different stiffnesses after treatment with XAV939. Data are means ± SD, **P < 0.01.

To assess the transcriptional activity of β-catenin after its nuclear translocation, a dual-luciferase reporter system (TOP/FOP) was used to detect its binding with TCF. We transfected DPCs with TOP flash (or FOP flash with a mutated TCF-binding site), which expressed GFP under the control of the TCF promoter. Significantly, the stiffer substrate promoted TCF transcription, as evidenced by GFP fluorescence intensity (Fig. 5B). Furthermore, the results of bioinformatics analysis showed that there were binding domains of TCF-1 in the promoters of *Osterix* and *Col1a1*, which had a positive function in DPC odontogenic differentiation (Fig. 5A; more detailed information in Supplementary Fig. S6). In addition, we confirmed that treatment with the β-catenin inhibitor (XAV939) reduced DPC mineralization in all stiffness groups (Fig. 5C).

## Discussion

Tooth loss or damage has been a prominent problem in human oral health. It is caused by dental caries, periodontal diseases, tooth agenesis or trauma [27–30]. Tooth tissue engineering based on tooth germ development is considered a promising strategy for replacing nonfunctional teeth [31]. This attractive concept is based on cell populations with regenerative potential. Therefore, we used DPCs, which were isolated from developing tooth germs. Besides the multipotency of DPCs, they are noninvasively and largely obtained from the third molars of humans, which are identified as a crucial stem cell resource in adults.

It has been demonstrated that stem cell differentiation is regulated by factors in the cellular microenvironment, such as physical cues and soluble chemical elements. Although previous studies have focused on effective growth factors in cell-based tooth tissue engineering, studies have increasingly focused on the mechanical properties of the cellular microenvironment over the past decades. In recent years, appropriate stiffness in the cell growing matrix has been demonstrated as a physical property of the cellular microenvironment regulating cellular behaviors, including migration, proliferation and differentiation [32–34]. A broad variety of cells derived from teeth have been reported to be influenced by substrate stiffness. Reportedly, the migration speed of dental pulp stem cells slightly increased as substrate stiffness decreased, which is associated with a reduction in FA size [35]. Proliferation of stem cells of apical papilla appeared better on stiffer substrates [36]. It was also confirmed that interface stiffness-mediated intercellular contacts in living human apical papillae through changes in gap junction tunnels [37]. The stiff matrix promoted osteogenic differentiation of stem cells from human exfoliated deciduous teeth [38]. Despite outstanding progress in designing an appropriate microenvironment for stem cell-based tooth regeneration, the mechanism by which substrate stiffness affects the odontogenic potential of stem cells remains unclear.

PDMS is a viscoelastic elastomeric bioassay platform for cell culture in cell mechanical studies. The properties of PDMS include biocompatibility, optical transparency, low toxicity, low cost, gas permeability and easy fabrication, which allow for cell culture and real-time observation [39, 40]. Accordingly, PDMS is widely applied to studying cell–cell and cell–substrate interactions and to detect biophysical signals. Some new cell-imprint surface modification techniques have been based on modified PDMS surfaces to control cell seeding into patterns for efficient cell-based studies. In particular, the elastic moduli of PDMS can be manipulated by changing the ratio of the curing agent to the oligomeric base, thereby providing a model of adjustable mechanical properties to investigate stiffness-dependent cell responses. Herein, we choosed the proportion of the curing agent and oligomeric base to 1:5, 1:15, 1:30 and 1:45, as our previous report. In our results, the range of elastic moduli was from 1.22 MPa to 30 kPa (Fig. 1D). It has been shown that stiff substrates with the elastic modulus >75 kPa significantly enhance dental pulp stem cell mineralization *in vitro* [41]. As our study was about mechanism in substrate stiffness-driven mineralization, the elastic moduli chosen in our report included above and below 75 kPa. DPCs were then cultured on various stiffnesses, and their phenotypes were analyzed. Notably, substrate stiffness changed the phenotype of DPCs. As shown in Fig. 1, the stiffer substrate promoted DPC extension, as evidenced by the expression of α-tubulin and β-tubulin. Although the apoptosis detecting results showed that DPCs appeared more survival in the intermediate stiffness (Supplementary Fig. S2B), the potential for DPC odontogenic differentiation was highest in the 1:5 group (the stiffest group). The stiff substrates provided exogenous stimuli to promote DPC differentiation from a stemness state, while DPC might show apoptosis for the sudden microenvironment changes on the stiffest substrate at the early stage.

To further explore the mechanism in the effect of stiffness on dentinogenesis, we analyzed the mechanical signal transduction initiated on the cell–substrate contacts, enabling changes in FAs. Paxillin is a FA protein and acts as a scaffold with multiple domains at sites of cell adhesions to the ECM for recruiting adhesion proteins and transmitting molecule signals [11]. Therefore, we focused on the role of paxillin in odontogenic differentiation of DPCs on stiff substrates. Figure 3 showed a higher expression level and more dotted distribution of paxillin in DPCs on stiffer substrates, indicating a stiff substrate-regulated intracellular pathway cued by paxillin. We then transfected adenovirus to overexpress paxillin in DPCs. Paxillin knockdown by siRNA downregulated the calcification of DPCs at all stiffness levels in this study. Thus, it was suggested paxillin was central to signal transduction during stiffness-driven DPC odontogenic differentiation.

The mechanotransduction from cell–matrix to cell–cell adhesions plays a regulatory role in cell differentiation [42], which is influenced by mechanical cues in the local cellular microenvironment [43]. Therefore, we investigated the interplay between cellular attachment to the substrate and intercellular interactions under the stimulus of mechanical stiffness. We focused on cadherin receptors, which are known to play a key role in cell–cell contacts. N-cadherin, also known as cadherin-2, represents a multigene family of Ca^2+^-dependent glycoproteins to mediate intercellular adhesions [15, 44]. Apart from their role in the formation of cell–cell contacts, cadherins have been known to participate in intracellular signaling cascades to modulate cell behaviors in various cell types. Overexpression of N-cadherin in cancer cells accelerates malignant cell migration and invasion [45]. Enhanced expression of N-cadherin promotes motility and invasiveness of epithelial cells [46]. N-cadherin is a prospective cell surface marker of human MSCs, possessing a pronounced ability for cardiomyocyte differentiation [47]. Cadherin-11 is localized to FAs, thus promoting cell–matrix adhesions [18]. Therefore, we speculated that N-cadherin in mesenchymal stem cells was the mediator of the mechanical force transition from cell–matrix interfaces to intercellular contacts. We hypothesized that the initiation of paxillin change at cell–substrate interfaces induced N-cadherin-mediated cell–cell contacts.

Cadherin protein forms protein complexes with β-catenin on the cell membrane, which is known as a vital player in signal transduction from the cytoplasm to the nucleus [48]. β-catenin expressed more in the nucleus after knockdown of N-cadherin (Supplementary Fig. S4). We speculated that paxillin could influence N-cadherin/β-catenin complex formation and β-catenin translocation into the nucleus, which mediated DPC odontogenic differentiation. The interaction between PXN and the E-cadherin (or N-cadherin)/β-catenin complex was experimentally determined by STRING analysis and text mining (Fig. 4A). However, the mechanism by which paxillin knockdown affects the distribution and expression of N-cadherin/β-catenin protein complexes remains unclear. In the present study, we silenced the paxillin gene using siRNA to detect changes in N-cadherin distribution. We provided evidence that paxillin knockdown slightly increased total N-cadherin protein expression (Fig. 4C). In addition, the IF results in Fig. 4B showed that N-cadherin expression increased mainly in the cytomembrane junctions of neighboring cells, which was the domain to which β-catenin binds. Therefore, we further detected β-catenin distribution in DPCs after paxillin expression decreased. As shown in Fig. 4D–F, nuclear β-catenin expression levels decreased with siPXN treatment, suggesting that β-catenin was a downstream molecule of paxillin in the process of stiffness-mediated odontogenic differentiation in DPCs. Because nuclear β-catenin activity facilitated TCF transcription, we performed a dual-luciferase reporter assay to compare TCF transcriptional levels in the stiffest (1:5) and softest (1:45) groups in this study, which assisted in determining β-catenin binding with TCF and their activity as transcriptional coactivators in the nuclei (Fig. 5B). Moreover, bioinformatics analysis showed that TCF-1 could bind to specific sites in the promoters of *Osterix* and *Col1a1*, contributing to DPC odontogenic differentiation (Fig. 5A). Following treatment with β-catenin inhibition (XAV939), DPC mineralization levels decreased in all PDMS groups with different stiffnesses (Fig. 5C). Thus, we concluded that N-cadherin/β-catenin ultimately participated in stiffness-mediated odontogenic differentiation. Our results may not only contribute dentinogenesis study but also provides some cues for other fields. It was reported increasing N-cadherin expression increased glioma stem cells stemness, and rendered their radioresistant [49]. According to our results, regulation of microenvironment stiffness might provide a new strategy for tumor therapy and paxillin was the upstream of N-cadherin/β-catenin in this mechanotransduction process.

In our study, the substrate stiffness-dependent adhesion force influenced intercellular contacts and ultimately regulated DPC odontogenic differentiation. Paxillin, which is responsible for cell–cell adhesions, is less known for its role in cell–matrix adhesions. We have demonstrated that paxillin-mediated surface stiffness cues modulated N-cadherin-mediated intercellular signal transduction. Because catenin proteins are associated with cadherins at cell–cell junctions [50, 51], we further provide evidence that the loss of paxillin decreased β-catenin translocation into the DPC nuclei, thereby reducing DPC mineralization. Thus, we identify the role of paxillin in cellular trafficking of N-cadherin/β-catenin signaling, which mediates DPC odontogenic differentiation.

## Conclusion

We investigated the effects of substrate stiffness on DPC odontogenic differentiation through the interplay of cell–matrix and cell–cell contacts. The stiffer substrates are beneficial to DPC odontogenic differentiation depending on cell adhesions. Mechanosignaling transduction initiated by paxillin then influenced N-cadherin-mediated cell–cell contacts and further regulated the accumulation of active β-catenin in the nuclei, resulting in stiffness-dependent DPC odontogenic differentiation. Our findings provide new insights into matrix mechanics-directed stem cell differentiation and a deeper understanding of the interplay between cell–substrate and cell–cell contacts in tissue engineering. The future work could be carried out using animal models toward finding the scaffolds with appropriate physical properties that are associated with tooth regeneration and stem cell-based therapy strategy.

## Supplementary Material

rbac100_Supplementary_Data
