# Supplementary material for: Paxillin tunes the relationship between cell–matrix and cell–cell adhesions to regulate stiffness-dependent dentinogenesis
Source: Regen Biomater. 2022 Dec 10;10:rbac100. doi: 10.1093/rb/rbac100 (PMC9847533; doi:10.1093/rb/rbac100)
Supplement: rbac100_Supplementary_Data [file rbac100_supplementary_data.docx]

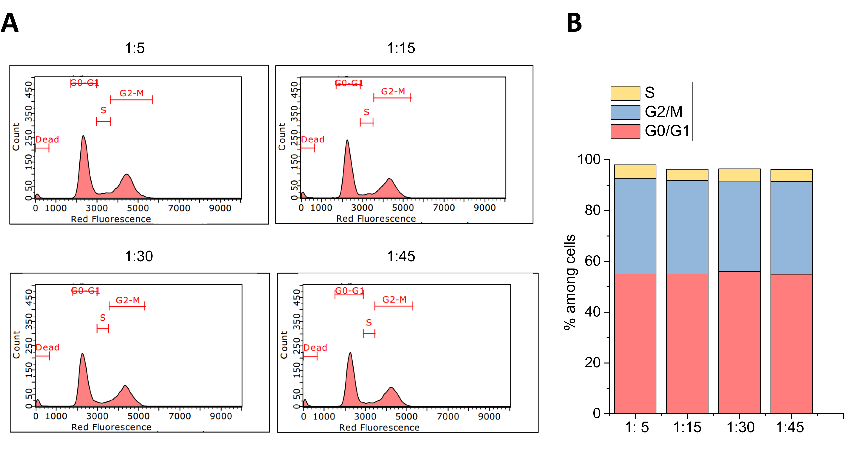


**Figure S1. DPC proliferation on PDMS substrates with different stiffness.** (A) Cell cycle was detected by flow cytometry. (B) Proportion of S stage, G2/M stage, G0/G1 stage.


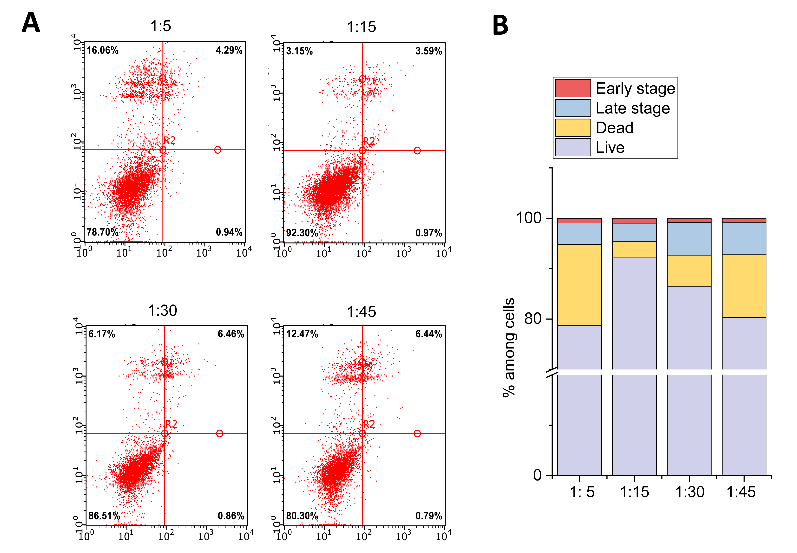


**Figure S2. DPC apoptosis on PDMS substrates with different stiffness.** (A) Apoptotic rate detected by flow cytometry (B) Proportion of normal, necrosis, early apoptosis, and late apoptosis in DPCs.


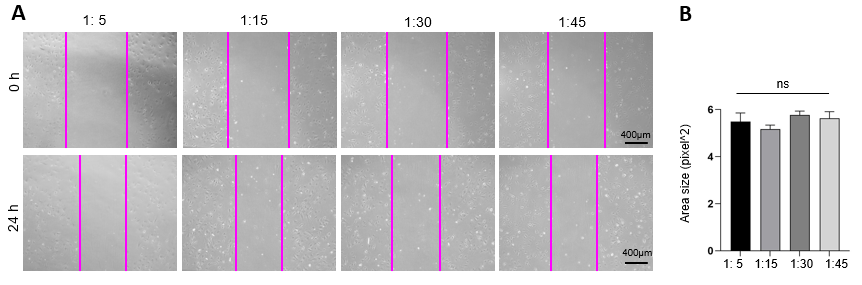


**Figure S3. DPC migration on PDMS substrates with different stiffness.** (A) Cell migration was detected by scratch method. (B) Quantification of cell migration area after 24h.


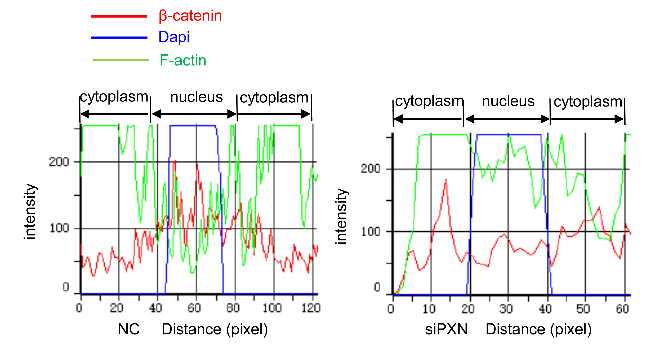


**Figure S4. β-catenin distribution was regulated by paxillin.**

Distribution of β-catenin. Green line represents F-actin. Blue line represents Dapi. Red line represents β-catenin.


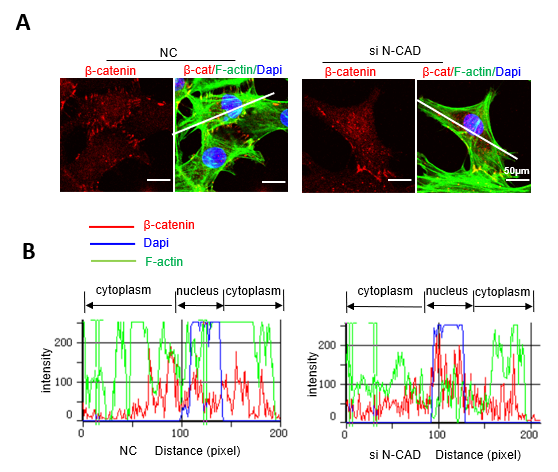


**Figure S5. β-catenin distribution was regulated by N-cadherin.**

1. β-catenin distribution after N-cadherin knockdown. (B) Distribution of β-catenin. Green line represents F-actin. Blue line represents Dapi. Red line represents β-catenin.


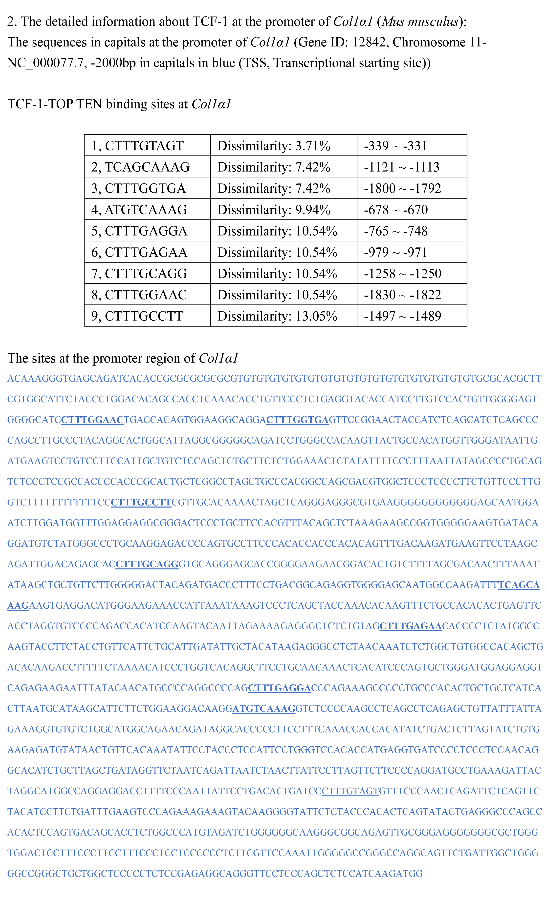

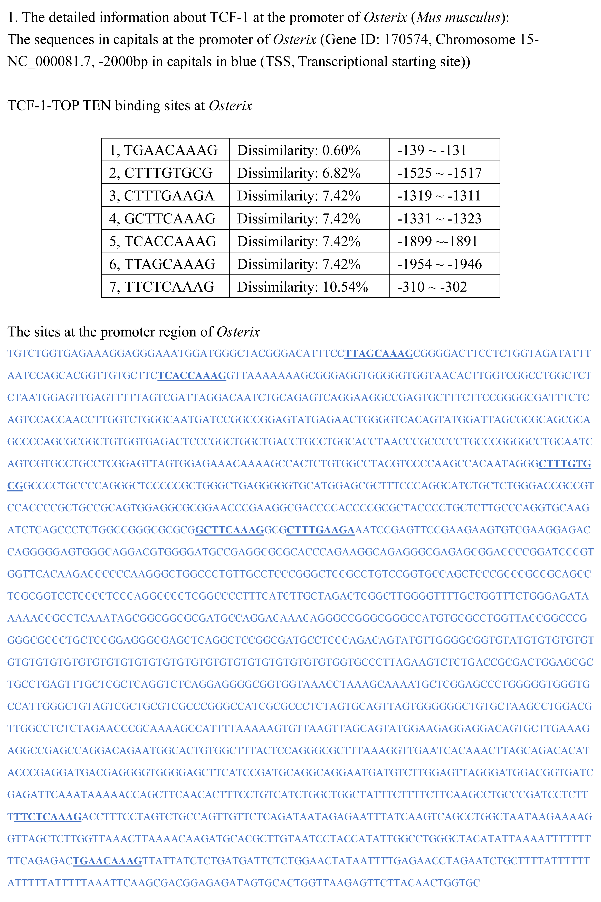


**Figure S6. The detailed information of about TCF-1 at the promoter of *Osterix* and *Col1a1*.**
